# Computational Models of Auditory Scene Analysis: A Review

**DOI:** 10.3389/fnins.2016.00524

**Published:** 2016-11-15

**Authors:** Beáta T. Szabó, Susan L. Denham, István Winkler

**Affiliations:** ^1^Faculty of Information Technology and Bionics, Pázmány Péter Catholic UniversityBudapest, Hungary; ^2^Institute of Cognitive Neuroscience and Psychology, Research Centre for Natural Sciences, Hungarian Academy of SciencesBudapest, Hungary; ^3^School of Psychology, University of PlymouthPlymouth, UK

**Keywords:** auditory scene analysis, computational model, auditory object representation, predictive processing, auditory streaming, bi-/multi-stable perception

## Abstract

Auditory scene analysis (ASA) refers to the process (es) of parsing the complex acoustic input into auditory perceptual objects representing either physical sources or temporal sound patterns, such as melodies, which contributed to the sound waves reaching the ears. A number of new computational models accounting for some of the perceptual phenomena of ASA have been published recently. Here we provide a theoretically motivated review of these computational models, aiming to relate their guiding principles to the central issues of the theoretical framework of ASA. Specifically, we ask how they achieve the grouping and separation of sound elements and whether they implement some form of competition between alternative interpretations of the sound input. We consider the extent to which they include predictive processes, as important current theories suggest that perception is inherently predictive, and also how they have been evaluated. We conclude that current computational models of ASA are fragmentary in the sense that rather than providing general competing interpretations of ASA, they focus on assessing the utility of specific processes (or algorithms) for finding the causes of the complex acoustic signal. This leaves open the possibility for integrating complementary aspects of the models into a more comprehensive theory of ASA.

## Introduction

In most situations, we receive sounds from an unknown number of different sources. The task of the auditory system is to parse the complex mixture in order to determine the likely sources of the incoming signals. In his groundbreaking book, Bregman (1990) termed this process *auditory scene analysis* (ASA). Although the incoming acoustic information does not fully specify the sources (at least not for the general case, when both the listener and the sources may move; Stoffregen and Bardy, 2001), everyday experience tells us that we can reliably decompose auditory scenes. That is, under natural circumstances, our auditory perception is rarely chaotic or misleading. However, the neural mechanisms by which the human (and animal) brain achieves this feat are largely unknown. In the past three decades, several theories have been postulated for explaining the processing of complex auditory scenes and the perceptual phenomena deemed to exemplify some crucial aspect of it. Many of these theories have been implemented in the form of computational models. Our aim here is to provide a theoretically motivated overview of recent models.

The most recent review discussing computational models of ASA was published in 2006 (Wang and Brown, 2006) (for a previous review, see Cooke and Ellis, 2001). Since then, some important new ideas have permeated the field (e.g., predictive processing and temporal coherence) and also some of the earlier models have been updated and extended. Therefore, we focus on the new theoretical developments and refer readers interested in the earlier models to the previous reviews (Cooke and Ellis, 2001; Wang and Brown, 2006). Some detailed reviews of ASA are available to the reader (Carlyon, 2004; Haykin and Chen, 2005; Snyder and Alain, 2007; Ciocca, 2008; Denham and Winkler, 2015) and we do not wish to reiterate them. Therefore, we will only introduce the most important phenomena, terms, and the main theoretical approaches providing basis for our review.

### Main theoretical approaches and issues

Bregman (1990) broke down ASA into two stages. In the first stage, incoming sounds are grouped in parallel by various heuristic algorithms, which are assumed to implement the Gestalt principles of perception (Köhler, 1947) (for a review specific to the auditory modality, see Denham and Winkler, 2015). These groupings then compete with each other in a second stage, with the winner emerging in perception. The outcome is a coherent succession of sounds, termed an *auditory stream*, which can be attended and manipulated by cognitive operations. Bregman (1990) distinguished auditory streams from perceptual objects, as a stream can combine contributions from several sound sources (e.g., the melody played by an orchestra). However, more recent definitions of auditory perceptual objects (Kubovy and Van Valkenburg, 2001; Griffiths and Warren, 2004; Winkler et al., 2009) include both representations of sound sources and sound patterns (such as a melody), because both can be used in mental operations. Auditory perceptual objects represent parts of the acoustic input that can be segregated from other objects. They describe the object by the perceptual features extracted from the input and are invariant with respect to irrelevant acoustic differences. They allow information from an object to be linked across time and possibly across modalities (such as between the speaker's lip movements and the speech sounds, whose congruence improves the intelligibility of speech in noise, while their incongruence may produce the McGurk effect; Erber, 1975; McGurk and MacDonald, 1976; Helfer and Freyman, 2005). Finally, object representations may generate predictions for upcoming sounds generated by the same source (Winkler et al., 2009). In this review, we regard auditory streams as perceptual objects. While theoretical clarity would require us to term candidate groupings as *proto-objects* (which can become the objects of auditory perception by winning the competition), for the sake of easier reading, we shall use the term object throughout, referring to proto-objects only when distinguishing between the two meanings.

Much of the experimental work on ASA has focused on Bregman's first stage (for summaries, see Bregman, 1990; Moore and Gockel, 2002, 2012; Carlyon, 2004; Ciocca, 2008). Bregman (1990) distinguished spectral/concurrent and temporal/sequential grouping processes. The former are responsible for grouping together elements of the incoming sounds present at the same time (Alain et al., 2002; Ciocca, 2008), using cues, such as harmonicity. The latter link together sounds separated in time to form temporal sequences. Grouping is assumed to be biased by the *old* + *new heuristic*: continuation of previously discovered groups are preferred over the emergence of new ones, leaving the sound elements that cannot be accounted for by the current representation(s) to initiate the formation of new objects (Bregman, 1990). However, when, as is typical, multiple continuous non-stationary sounds are mixed together (e.g., Teki et al., 2011), the two kinds of grouping processes cannot be easily separated. Shamma et al. (2011, 2013) suggested that grouping occurs on the basis of temporal coherence between featural (e.g., spectral) constituents of the complex acoustic input. That is, similarly to the Gestalt principle of “common fate,” those parts of the input that recur together belong together. According to temporal coherence theory, a single process binds together both concurrent and temporally separate sound elements.

Bregman's second processing stage, the competition between objects, has received far less treatment. In most models, it is only implicitly assumed (e.g., Snyder and Alain, 2007) or it is not present at all (Shamma et al., 2011, 2013). There is, however, some experimental evidence supporting the existence of multiple processing phases in auditory stream segregation (Winkler et al., 2005; Snyder et al., 2006). Further, the existence of *bi- and multi-stable auditory perceptual phenomena* (i.e., when perception of the same stimulus switches back and forth between two or more interpretations, respectively; Schwartz et al., 2012) suggests that alternative descriptions of the auditory scene may be simultaneously represented in the human brain. (For generality, we will use the term multi-stability throughout the review).

Bregman (1990) intended his description of auditory scene analysis as a theoretical framework. As a consequence, some of the processes are underspecified. One question is whether competition occurs between objects or between coalitions of objects (termed *perceptual organizations*). For a discussion of this issue, see (Winkler et al., 2012). Another question is whether competition is continuous or only occurs when a new object is formed. Traditional descriptions of ASA assumed that for unchanging stimulation, after a period of evidence gathering, the dominant (perceived) object is established once and for all. Thus there is no need for competition to continue until the stimulation changes. However, results from experiments using multi-stable stimulus configurations (Roberts et al., 2002; Denham and Winkler, 2006; Denham et al., 2013; Pressnitzer and Hupé, 2006; Schadwinkel and Gutschalk, 2011) suggest that competition between the alternatives is continuous even for unchanging stimuli.

One can also ask what fuels the competition between objects. The initial activation may be derived from the grouping processes (i.e., some measure of how easily the elements of an object could be linked). However, theories assuming continuous competition must also consider further effects on object strength (e.g., competition itself is typically modeled by mutually inhibitory interactions; Leopold and Logothetis, 1999). Several recent theories emphasize the predictive nature of perception (Gregory, 1980; Friston, 2005; Bar, 2007). If objects were represented as generative models they could be tested against the actual input, which would provide a continuous evaluation of their validity and a source of activation, as well as the possibility to eliminate outdated representations.

Another area in which Bregman's theoretical framework requires further elaboration is regarding the nature of the memory representations underlying ASA. Two issues, possibly two forms of memory, can be considered: one allowing discrete sounds to be linked and another representing the resulting temporal sound pattern. Results from studies of stimulus-specific adaptation (SSA) suggest that the upper bound of the first effect is ca. 2 s (Ulanovsky et al., 2003, 2004). Unlike this shorter-term effect, the memory encoding the pattern that defines a sound object may be brought into consciousness and/or enter various mental operations over much longer time periods. Based on experimental results of auditory deviance detection, Winkler (2007) suggested that sound sequences are represented by the characteristic relationships between adjacent sounds (*transitional probabilities;* cf. Mittag et al., 2016). Transitional probabilities are conducive of predictive processing, because they allow the system to predict the most likely continuation of a sequence. Shamma et al. (2011, 2013) temporal-coherence based explanation does not require the assumption of different forms of memory. Instead, temporal coherence is established in parallel on multiple time scales and is a function solely of the stimulus itself (for compatible experimental evidence, see O'Sullivan et al., 2015). However, it is not easy to see, how this system would encode longer temporal patterns, such as melodies. The presence of separate patterns/melodies is known to allow interleaved sound sequences to be segregated (e.g., Dowling, 1973; Bey and McAdams, 2002; Bendixen et al., 2010) even in the absence of additional cues (Szalárdy et al., 2014).

### Stimulus paradigms used for studying ASA

**Figure 1:**
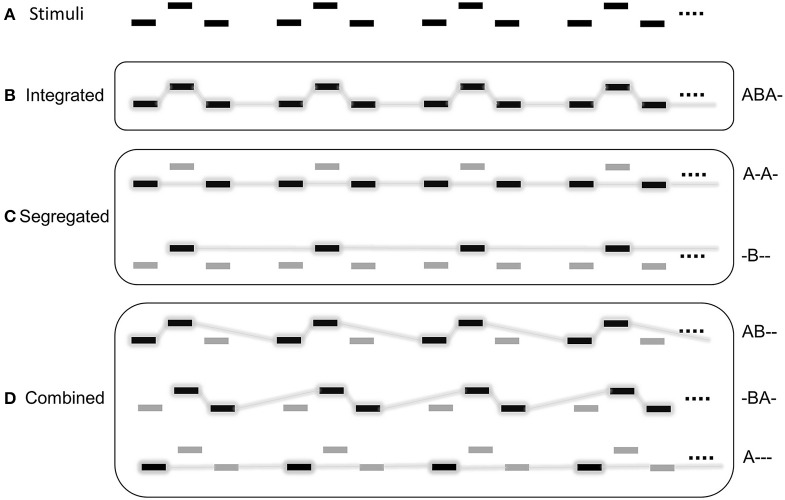
**Schematic depiction of the auditory streaming paradigm (A)** and its possible perceptual interpretations grouped into 3 categories **(B–D)**. Rectangles depict the “A” and “B” tones. Sounds perceived as part of the same stream are connected by lines in the lower panels **(B–D)**. Darker notes with gray background indicate the stream in the foreground (also described with symbols to the right of each of the lower panels; **B–D**). Reprinted with permission from Farkas et al. (2016b).

The most widely studied stimulus paradigm within the context of ASA was introduced by van Noorden (1975): the *auditory streaming paradigm* consists of a repeating tone triplet of the form ABA- (where “A” and “B” denote two different tones and “-” stands for an interval equaling the common duration of “A” and “B;” Figure 1A). This stimulus can be primarily heard either in terms of the ABA tone-triplets (producing a galloping rhythm; termed the *integrated percept*; Figure 1B) or as two separate isochronous streams: a faster paced one consisting of the “A” tones and a slower one consisting of the “B” tones (termed the *segregated percept*; Figure 1C). Other repeating patterns (such as AB—and–A-; together termed the *combined percept*; Figure 1D) can also be experienced, albeit typically with lower incidence (Denham et al., 2014). Based on psychophysical testing of the parameter space of frequency difference and presentation rate, van Noorden (1975) established three regions. In the *integrated region*, listeners could not hear the sequence as segregated, whereas in the *segregated region* they could not hear it as integrated. Large frequency differences between the two tones and fast presentation rates increased the likelihood of segregation, whereas small differences and slow stimulus presentation rates favored integration. With parameters falling between the integrated and the segregated regions, listeners could voluntarily bias attention in favor of integration or segregation (termed the *ambiguous region*). The boundaries between the ambiguous and the other two regions were termed the *fission* and *temporal coherence boundaries*.

Most classical studies presented trains of 5 to 20 s duration. The listener's perception was tested at the end of the train. Some more recent studies presented longer (>1 min) sequences and asked listeners to continuously report their perception. These studies showed that perception does not settle on one of the alternative organizations. Rather, it switches back and forth between the alternatives even for parameters strongly promoting one of them (perceptual multi-stability; e.g., Roberts et al., 2002; Denham and Winkler, 2006; Pressnitzer and Hupé, 2006; Denham et al., 2013; Schadwinkel and Gutschalk, 2011). This phenomenon is characterized by a sequence of *perceptual phases*, which are intervals during which perception remains constant. The first *perceptual phase* is typically longer (>10 s) and it is more affected by stimulus parameters than the subsequent phases (Pressnitzer and Hupé, 2006; Denham et al., 2013). Further, in contrast to the classical notion that perception of a new stimulus starts out by integrating all sounds into a single coherent percept, some studies have found that segregation can also appear as the first reported percept (Deike et al., 2012; Denham et al., 2013). The temporal pattern of perceptual switching appears to be similar to that observed for visual multi-stable phenomena, such as binocular rivalry (Pressnitzer and Hupé, 2006; Hupé and Pressnitzer, 2012; Kondo et al., 2012). Similarities include the inevitability of switching, approximately log-normal distribution of phase durations (Pressnitzer and Hupé, 2006; Farkas et al., 2016b), and characteristic individual switching patterns (Denham et al., 2014; Farkas et al., 2016b). However, the initial observation that successive perceptual phase durations are largely uncorrelated (Pressnitzer and Hupé, 2006) has been recently questioned (Barniv and Nelken, 2015).

The processing of spectral/concurrent cues of ASA is most often studied by manipulating one partial of a *harmonic complex tone*. Such stimuli are usually perceived as two concurrent sounds: a complex tone with the same pitch as the original harmonic complex and a separate pure tone corresponding to the manipulated partial (Moore et al., 1986; Hartmann et al., 1990; Darwin et al., 1995; Alain et al., 2001). This is because frequency components in harmonic relationship (integer multiples of a common base frequency) are grouped together and perceived in terms of a single pitch (that of the fundamental), thus allowing them to be discriminated from tones and harmonic complexes with different fundamental frequencies (Rasch, 1978; Duifhuis et al., 1982). The most common manipulations are mistuning (i.e., the frequency of the partial is increased or decreased), and delaying or delivering a partial from a different location than the rest (e.g., McDonald and Alain, 2005; Lipp et al., 2010; Kocsis et al., 2014). Greater amounts of manipulation, manipulation of the lower as opposed to the higher harmonics, multiple manipulations of the same harmonic, and congruent manipulation of two or more harmonics increase the likelihood that two concurrent sounds will be perceived (McDonald and Alain, 2005; Lipp et al., 2010; Kocsis et al., 2014).

**Figure 2:**
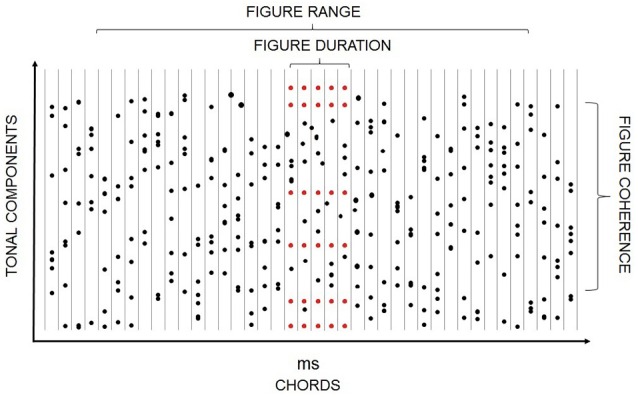
**Schematic illustration of a stimulus including a “figure” component**. Black dots depict random tonal elements while red ones represent repeating ones. Chord onsets are represented as vertical lines. The x axis shows both time and the serial position within the stimulus. The y axis provides a qualitative representation of frequency. Figure duration (the number of repeated tone complexes), figure coherence (number of tonal components comprising the repeated tone complex), and the range within which the figure can appear are marked.

Most everyday sound sources emit series of complex sounds and so require the auditory system to jointly utilize concurrent and sequential cues. The encoding of such temporal patterns is often studied using *tone clouds*, which consist of a large number of pure tones of random frequencies. Tone clouds have been used in information masking designs measuring the effect of auditory stream segregation on detecting tone repetition within a protected frequency range of the cloud (e.g., Kidd et al., 1994; Elhilali et al., 2009b; Akram et al., 2014a). They also allow the creation of target patterns for detection (e.g., Kumar et al., 2014; Barascud et al., 2016) as well as variable backgrounds within which repeating target patterns can be detected. For the latter purpose, Teki et al. (2011) created a sound configuration consisting of a series of tonal complexes composed of random frequencies, which were presented without pause (Figure 2). Within a continuous part of this stimulus, a subset of the tonal elements is repeated. The repeated tonal complex can then be perceived as a “figure” over the background of the randomly varying chords. Increasing the number of repeating frequencies or the number of repetitions increases the likelihood of segregation (Teki et al., 2011; Tóth et al., 2016). Because the “figure” is created by manipulating the temporal coherence of a part of the stimulus (i.e., by repeating some tones while the rest of the tones are randomly varied), the temporal coherence theory of ASA provides a good explanation for figure-ground segregation in these cases (Teki et al., 2013). Teki et al. (2016) extended their original finding by showing that repetition of the tonal complex is detected even when the tone cloud and, within it, the repeating complex is interspersed with white noise segments, thus making the figure acoustically non-continuous. Connecting non-adjacent segments into a coherent stream has been previously observed for temporal/sequential grouping (Bendixen et al., 2012). The generality of this feature across different forms of auditory stream segregation speaks to the robustness of this important auditory function.

### Measures of ASA

Behavioral measures of ASA can be divided into two classes: subjective and objective measures. Subjective measures require the listener to report their perception, while objective measures have listeners perform a task whose difficulty depends on the perceptual organization of the incoming sounds. As a typical subjective measure of the auditory streaming paradigm, van Noorden (1975) asked listeners to tell whether they heard a galloping rhythm as this shows that the listener experienced the tone sequence as integrated. In the same paradigm, temporal order judgments can serve as an objective measure of segregation, because it has been found that listeners perform this task much better when the two target sounds belong to the same stream than when they belong to two different streams (Bregman and Campbell, 1971). The obvious disadvantage of subjective measures is that they cannot be directly validated. However, Farkas et al. (2016a) showed that when the instructions are carefully formulated and the listener's understanding of them is monitored by catch trials, then valid perceptual data can be obtained from subjective reports in the auditory streaming paradigm. The advantage of subjective measures is that they allow monitoring the listener's perception of a sound sequence at a far better temporal resolution than objective ones. In contrast, objective measures only allow the listener's perception to be assessed at much less frequent discrete time points. Further, requirements of the objective-measure task may cause listeners to favor one perceptual organization over another. A common problem of the two measurement methods is that experimenters typically limit the percepts to be considered. Brain responses provide an alternative to behavioral measures. Their main advantages are that they (1) do not necessarily need the listener to perform some task with the sounds, and (2) provide information about the neural bases of ASA. However, they suffer from some of the same problems that have been described above, because they need to be validated by behavioral measures. To date brain measures have seldom been used for validating computational models of ASA, therefore we do not review them here (for some of the neuroscience methods used for studying ASA, see the next section as well as Fishman et al., 2001; Bee and Klump, 2004; Deike et al., 2004; Gutschalk et al., 2005; Wilson et al., 2007; Alain and Winkler, 2012; Teki et al., 2013; O'Sullivan et al., 2015; Teki et al., 2016).

### What do we know about the neural bases of ASA?

The cochlea decomposes the signal into a set of frequency components and establishes the tonotopic organization that is found throughout much of the auditory system up to and including the primary auditory cortex; for an overview of the subcortical auditory system see Irvine (2012). However, even at this early stage, processing is not just a passive feedforward process. For example, cochlear intrinsic nonlinearities increase the saliency of onsets, emphasize spectral peaks and reinforce harmonically related components in incoming signals. Recurrent feedback, primarily mediated via physical changes in outer hair cell motility, provides a mechanism for adaptive gain control and active modulation of cochlear processing (Guinan, 2006). As the signals pass onwards from the cochlea toward the brain, additional features are extracted and represented in overlapping maps, largely in parallel across the tonotopic axis; such features include onsets, offsets, periodicities, amplitude and frequency modulations (AM, FM), and interaural time and level differences (ITD, ILD). Together these features form the basis for the grouping processes underlying ASA.

Subcortical processing provides cortex with time-locked information about acoustic features detected within the incoming mixtures of sounds, but this information is agnostic with regard to which features belong together or the sources from which they might originate. Cortex then, possibly through inferential processes (Friston, 2005), groups and segregates features into composite event and object representations; representations which become increasingly more abstract at higher levels of the auditory processing hierarchy (Kumar et al., 2014). Thus it is likely that cortex is responsible for object formation. Similarly to other sensory systems, the cortical auditory system is organized in a hierarchical manner (Leaver and Rauschecker, 2010). For example, a pitch processing hierarchy runs from primary auditory cortex in Heschl's gyrus through planum temporale, superior temporal gyrus and planum polare (Patterson et al., 2002). Differential activations along this pathway distinguish sounds from silence, pitched from unpitched sounds, and melodic patterns from repeated pitches. Further, activity along this pathway also correlates with the emergence of categories (e.g., voices, musical instruments) from feature combinations (Leaver and Rauschecker, 2010). Consistent evidence comes from magnetoencephalographic (MEG) studies of cortical responses to events in speech mixtures (Simon, 2015): primary auditory cortical activations with latencies around 50 ms are primarily related to feature-based representations, while those localized to planum temporale with latencies from 100 ms onwards are related to object-based representations (see also Näätänen and Winkler, 1999).

Evidence for cortical feature grouping has been demonstrated by an electrophysiological study of onset and offset responses in single neurons in primary auditory cortex (A1) (Scholl et al., 2010). It was shown that onset transient and offset transient inputs were driven by different synapses, suggesting that onset and offset signals from different subcortical populations converge onto individual A1 neurons, which then produce a composite response. Thus these cortical cells may effectively perform the temporal boundary grouping role proposed by Ciocca (2008). Grouping by harmonicity also appears to depend on cortical processing, and is associated with a cortical pitch onset response occurring at a latency of 100–150 ms (Krumbholz et al., 2003) and a more sustained pitch response (SPR) which gradually builds up and then remains roughly constant for the duration of the pitched sound (Gutschalk et al., 2007). As for grouping by sequential cues, investigations of the neural correlates of auditory streaming have given rise to the suggestion that the build-up of streaming can be explained by a combination of feature selectivity, forward suppression, and multiscale adaptation (Fishman et al., 2004; Bee and Klump, 2005; Micheyl et al., 2005). For example, Fishman et al. (2001, 2004) found that differential suppression of the responses to tones differing from the best frequency of the cells in monkey primary auditory cortex took time to develop. The effect depended on the presence of alternating best frequency and non-best frequency tones as well as on the presentation rate and the frequency difference between the best frequency and the non-best frequency tones. These findings could account for the build-up of stream segregation (Micheyl et al., 2005). However, the above mentioned studies mainly focused on short-term changes in neural responses to alternating tones and did not consider the possibility of multi-stability. Using longer sequences that produce multi-stable perception, both transient activity in response to perceptual switches and sustained responses that correlate with the experienced perceptual organization have been demonstrated (Kondo and Kashino, 2009; Schadwinkel and Gutschalk, 2011). Sustained responses in auditory cortex appear to encode perceived repetition rates and increase in response to segregation. Transient responses synchronized with perceptual switching have been found in auditory cortex (Kondo and Kashino, 2009; Schadwinkel and Gutschalk, 2011), thalamus (Kondo and Kashino, 2009), inferior colliculus (Schadwinkel and Gutschalk, 2011), and intraparietal sulcus (Teki et al., 2016). Although it is not clear how or where switching is triggered, these studies provide evidence for a tight interaction between cortical and subcortical processing in resolving the ASA problem. Further, some brain imaging studies in humans found that correlates of auditory stream segregation (2 vs. 1 object) were evident in parietal cortex (Cusack, 2005). Thus it is likely that the full network underlying the formation of auditory perceptual objects extends beyond the auditory system.

Repetition is a powerful grouping and segregation cue, the effects of which can be demonstrated even in the absence of other cues (McDermott et al., 2011; Teki et al., 2011, 2013, 2016). Sensitivity to repetition (in the presence of a variable background) may underlie the robustness of perception with regard to natural variability in signals, such as speech. In support of this idea, cortical responses to a target speaker have been shown to be invariant with respect to irrelevant object details (such as intensity changes) (Ding and Simon, 2014; Simon, 2015). By studying transitions between random and patterned tone sequences it has been shown that steady-state responses in auditory cortex, inferior frontal gyrus, and hippocampus correlate with the predictability of the sequence (Barascud et al., 2016; Kumar et al., 2016). That is, the sustained response increases with increasing predictability, suggesting that this representation is precision weighted as opposed to being a result of adaptation or a correlate of prediction error, both of which would suggest a response decrement (Mathys et al., 2011; Teki et al., 2016). Offset responses to auditory objects (patterned sequences) are compatible with the notion of predictive auditory object representations (Andreou et al., 2015; Barascud et al., 2016), with some caveats; offsets responses to repeating pitch patterns require about three violations (Barascud et al., 2016), and offset responses to repeating temporal patterns are only elicited by isochronous sequences, whereas offset responses to non-isochronous sequences seem to require attention (Andreou et al., 2015). In summary, while neural signatures have been found for most of the important perceptual effects of ASA, exactly how separable object representations are instantiated in cortical networks is still largely unknown.

## Modeling auditory scene analysis

Computational models of auditory scene analysis vary in their fundamental goals; while some attempt to address the complexity that the auditory system faces when processing realistic sounds (such as speech; Nix and Hohmann, 2007; Elhilali and Shamma, 2008; Krishnan et al., 2014; Thakur et al., 2015) in natural environments, others (Wang and Chang, 2008; Boes et al., 2011; Mill et al., 2013; Barniv and Nelken, 2015; Rankin et al., 2015) are built in order to test the potential of some algorithm to simulate specific behavioral and/or neurophysiological experiments. For example, Wang and Chang (2008) measure the fitness of their model based on its ability to reproduce the fission and temporal coherence boundaries reported by van Noorden (1975). In general, models of this type can be evaluated by the degree to which they can replicate behavioral or neurophysiological data (such as the validating metric in Goswami et al., 2011). In contrast, models built to process realistic auditory scenes are typically evaluated using the signal to noise ratio of their output or a measure of similarity between the spectrograms of the output signal and that of the original unmixed target sounds (e.g., the speech segregation by the model of Krishnan et al., 2014). The main issue distinguishing the two types of models is that those dealing with realistic scenes need to include feature extraction and binding, whereas those processing simplified inputs usually assume pre-processing stages acting to generate the required inputs to the model.

One can also categorize computational models based on the modeling principles they adopt. Here we distinguish between three broad classes of principles: (a) Bayesian inference rules, (b) neural processing, and (c) temporal coherence. The term neural here refers to the group of models that have been formulated with a view toward neurocomputational processes. Although this categorization does not follow *a priori* theoretical distinctions, we found that it determines some properties of the models to such degree that it makes sense to discuss models in these groups. In the rest of this review we will focus on relating existing computational models of ASA to the theoretical issues outlined in the previous sections. As all the models we review attempt to replicate human perception, we adopt a terminology that equates model responses to perceptual responses. A *sound event* is considered to be a discrete isolated sound with a beginning and an end (e.g., a single tone). A *perceptual event* is the response to that event within perceptual (model) awareness. A *proto-object* is a candidate grouping of perceptual events, and a *perceptual object* refers to a proto-object that emerges into perceptual awareness. For simplicity, we use the generic term *object* to refer to the perceptual representation that supports decisions about the likelihood that it generated an incoming event. We adopt the term *perceptual organization* to refer to the decomposition of the sonic environment into compatible groups of objects. In this terminology, the term *stream* proposed by Bregman (1990), and used in many of the papers reviewed here, can refer to a single object in the case of integration, or to two objects in the case of segregation.

The first issue we consider is whether the models include some form of competition between the representations of alternative objects or perceptual organizations. We first discuss models that consider more than one alternative for representing the input, with competition between them. In essence, these models follow the empiricist tradition (Helmholtz, 1860/1962), which assumes that the sources (causes) of the acoustic input are underspecified and the brain has to provide constraints for disambiguation. We then turn to models that assume the incoming sounds carry sufficient information for the brain to extract their causes in a single pass–a theoretical approach akin to that of J. J. Gibson and his followers (Gibson, 1979). Another central issue of this review is the extent to which the models utilize predictive processing. As mentioned in the introduction, recent theories (e.g., Gregory, 1980; Friston, 2005; Bar, 2007) regard perception as inherently predictive. Models predicting upcoming sounds are in principle capable of self-validation, thus potentially offer more robust performance. This would, of course, require that some measure of the success of the model—measured in terms of how well it can predict future sound events—is used to modify its representations either by direct feedback or through an error signal handled by higher-level processes in a hierarchical system (as proposed by Friston, 2005). Models differ considerably in their flexibility. Although they all aim to model ASA in general, some models are restricted to two object or foreground-background solutions, while others rely on a fixed pre-training phase. The extent to which the models are able to create, maintain and evaluate object representations dynamically is therefore an important distinguishing feature. Finally, most models address some specific issues, such as replicating perceptual switching patterns in response to multi-stable stimuli. These will also be noted and discussed.

As mentioned in the introduction, we selected models for this review which have been published since the last two reviews of computational models of ASA (Cooke and Ellis, 2001; Wang and Brown, 2006). Further, we only included models, whose focus was on ASA. That is, we do not discuss saliency detector models (such as De Coensel and Botteldooren, 2010; Oldoni et al., 2013) or models using some additional “external” cue to extract sound patterns from the background (such as Boes et al., 2011; Akram et al., 2014b). These models may be construed as descriptions of the effects of attention on ASA, rather than models of ASA *per se*. For example, Akram et al. (2014b) model is based on a variant of the temporal coherence model (which will be reviewed in full) aiming to test how an external attentional cue helps to select a single sound stream from a complex scene, whereas the model of Boes et al. (2011) determines the direction of possible sound sources, which could be used to direct attention, but does not attempt to group or segregate objects. Thus we will focus on models providing a description of how sound elements are grouped and separated from each other based solely on the sound input and possible prior training, where applicable. In the following sections, first Bayesian, then neural, and finally temporal coherence based models are discussed. The models reviewed are listed in Table 1.

**Table 1:** **The models reviewed together with their categorizations with respect to the main issues discussed in the review**.

**References**	**Competition**	**Prediction**	**Theoretical basis**	**Number of objects**
Barniv and Nelken, 2015	yes	yes	Bayesian	unlimited
Nix and Hohmann, 2007	yes	yes	Bayesian	2
Wang and Chang, 2008	yes	no	Neural	2
Pichevar and Rouat, 2007	yes	no	Neural	2
Mill et al., 2013	yes	yes	Neural	unlimited
Rankin et al., 2015	yes	no	Neural	3
Krishnan et al., 2014	no	no	Temporal Coherence	2
Ma, 2011	no	no	Temporal Coherence	2
Elhilali and Shamma, 2008	no	yes	Temporal Coherence	2

### Bayesian models

The models that use Bayesian inference all exploit predictive mechanisms. In these models, the acoustic environment is described by state vectors estimated from the input. Competition is mediated through the adjustment of priors: i.e., the current decomposition can affect the a-priori probability of other objects. As a consequence, in these models, prediction is strongly linked to competition. Although the role of the priors varies among models, the a-priori probabilities always represent predictions, even when they are based on prior training (as in the model of Boes et al., 2011) rather than on the current state of the model (as in Barniv and Nelken, 2015).

The models differ in the perceptual problem they address. Barniv and Nelken (2015) aim to simulate behavioral results obtained in auditory streaming experiments. In contrast to previous studies (Pressnitzer and Hupé, 2006), they found that the durations of successive perceptual phases were correlated and argued that this correlation reflects an evidence-accumulation process continuously operating in the background: long perceptual phases allow alternative objects to become stronger, which leads to a longer perceptual phase after the perceptual switch. Their model was formulated to account for this effect. Thus it uses a simplified tokenized input (i.e., simple tones represented by their frequency and timing).

The model of Nix and Hohmann (2007) tracks sound sources in space and segregates them from other sounds based on directionality. The authors used broadband sounds for training, and trained their model on exclusively spectral information (but not by the direction of the sound source).

#### Objects, sound organizations, and model dynamics

Although the term object is not explicitly used in these models, the concept, as we have defined it, is applicable. In Barniv and Nelken (2015) model each incoming sound event is assigned to a class; a class is analogous to an object. A Bayesian algorithm determines this assignment; the posterior probability of a given event belonging to class *k* is a function of the conditional likelihood of the event given that class *k* is the object that generates the input, and by the a-priori probability that class *k* occurs. The number of classes is not fixed in this model, and if the class probability of an incoming event is low then a new class is defined. When the model was tested on auditory streaming sequences it dynamically produced a sequence of one or two class solutions; the one class solution corresponding to integration, and the two class solution to segregation.

In Nix and Hohmann's (2007) model, the acoustic environment is represented by a state-vector. Objects are described in the state-vector by their short-term spectrum and direction (azimuth and elevation). Thus a state vector is the equivalent of a sound organization as defined in the introduction. The features of an object can be extracted from the state vector using the index value assigned to it (i.e., each value indexes the features of one object). The goal of the algorithm implemented by the model is to determine the state vector's posterior probability, i.e., the conditional probability of a given state vector given the acoustic observations. The number of possible objects repres–ented in the state vector, i.e., the number of the incoming voices was pre-specified (set to two in the actual implementation).

#### Prediction, competition and model dynamics

The model of Barniv and Nelken (2015) implements competition between alternative decisions on class membership by dynamically adjusting the priors (i.e., the probability of occurrence of each class). Upon the arrival of a new sound event the priors corresponding to the existing classes are changed. (In the event that the class probability of the incoming event is small, a new class is generated with a new prior). The change is proportional to the conditional likelihood of the sound event given that it was produced by the class. Thus evidence accumulation is encoded in the changing of the priors: evidence increases for classes that are likely to have generated the input, while the priors of the other class(es) are decreased.

In Nix and Hohmann's (2007) model, during the training phase, the priors and transitional probabilities of state vector coordinates that represent the short-term spectra of the objects are calculated, while those for the directional information are approximated by Gaussian functions. So the model requires prior knowledge of the sound sources to be tracked. During simulations on new input, the goal is then to determine the most likely state-vector distribution. For this purpose, the model defines *particles*, which consist of a state vector at a given time point and a weight. These particles represent a possible sound environment at a given time, i.e., the spectral and directional information of the two objects. The task is to filter out the state vectors which are not likely to match the input. The particle filter algorithm is realized by a Sequential Monte Carlo (SMC) method. Each particle predicts the next state vector. These predictions are tested against observations and the weight associated with each particle is updated according to its predictive value. Thus particles compete for predictive success. As the state vector contains information on all objects, the competition between particles can be regarded as a competition between sound organizations.

#### Output and model evaluation

The output of Barniv and Nelken (2015) model is a time series of discrete states describing whether the model assigns all sounds to a single class (integrated organization) or it sorts them into two classes (segregated organization). In the latter case, it does not say which of the classes would appear in the foreground. The authors compared the output of their model with human perceptual data from the auditory streaming paradigm; both their own data and data from Hupé et al. (2008). The model qualitatively replicated the positive correlation between successive perceptual phase durations found in the empirical studies.

Nix and Hohmann's (2007) model was tested for its ability to track and segregate one or two voices in a binaural mixture. The authors reported that the model could segregate voice envelopes after convergence (which took on the order of 50 ms of data), and that improvements in signal-to-noise ratio (SNR) in the region of 2–8 dBs could be achieved for input mixtures with 0dB SNR. Convergence was faster and more frequent when there was only one voice to track, and when the variability of the azimuth was low.

### Neural models

Common to models in this section is the representation of objects by units, the properties of which are inspired by neurons or networks of neurons. Thus the computations within these models could in theory be performed by neuronal networks, although none of the models claim that the networks they specify correspond to actual networks located in the brain. The strength of the activation of the object units determines the output of the model. Competition between objects is implemented through interactions (typically inhibitory) between the units. In general, these models use tokenized input. All models of this section were evaluated by comparing simulations to behavioral evidence.

Wang and Chang's (2008) model represent different frequency channels in terms of the activations of neural oscillators. While other models, such as the one of Wrigley and Brown (2004) implement a one-dimensional bank of oscillators, in Wang and Chang's model the oscillators are organized on a two-dimensional map, where the second dimension is time represented via delay-lines. The dynamics of the model is determined by local excitatory, global inhibitory connections between the oscillators. Wang and Chang's (2008) goal was to faithfully reproduce the three regions (integrated, segregated, and ambiguous) found by van Noorden (1975) for the frequency-difference/presentation-rate parameter space in the auditory streaming paradigm, but does not simulate more recent findings of perceptual multi-stability (e.g., Denham and Winkler, 2006; Pressnitzer and Hupé, 2006; Denham et al., 2013). The model uses tokenized pure tones as input sound events, represented by the frequency and the time of arrival of the event.

The model of Pichevar and Rouat (2007) aims to segregate speech from environmental sounds (e.g., siren, telephone ring) and is implemented in the form of a two-layered neural network. The first layer consists of oscillators organized into a two-dimensional space, with similar dynamics to the model of Wang and Chang (2008). Although the dynamics are similar, as they are governed by local excitation and global inhibition, binding in this model is based instead on temporal correlations between frequency channels, which is a principle widely exploited by temporal coherence models (reviewed in the next section).

Mill et al. (2013) explicitly used the concepts of proto-objects and objects described earlier; however, for simplicity we will only use the term object here. Objects are represented in the model as linked events. Each object, once formed, is represented by a coupled excitatory and inhibitory population of neurons that interact with other object representations through network connections that determine the dynamic behavior of the model and simulate the emergence of dominant perceptual organizations and switching between them through competition between objects. Note that the objects in this model represent candidates for perception that may not necessarily emerge into perceptual awareness. The model was formulated to capture the dynamics of perceptual multi-stability observed in the auditory streaming paradigm. Similar to Barniv and Nelken (2015), it does not assume a fixed number of objects, and construction of new object representations as well as competition between existing objects occurs continuously, with the dominant (perceived) object(s) stochastically changing even for an unchanging pattern of stimulation, as found in human experiments (e.g., Denham et al., 2013). The model employs predictive mechanisms in two ways: firstly to validate the representations of candidate objects extracted from the incoming sequence of sounds (object representations incorrectly predicting upcoming sounds are pruned) and secondly to instigate competition between objects (objects predicting the same sound event mutually inhibit each other). This model uses simplified tokenized input, similar to the model of Wang and Chang (2008) (i.e., simple tones represented by their frequency and timing parameters).

Similarly to the model of Mill et al. (2013), Rankin et al. (2015) aimed to model perceptual multi-stability in the auditory streaming paradigm (see Stimulus Paradigms Used for Studying ASA). However, they only modeled the dynamics of perceptual switching between the integrated and the segregated sound organization following the build-up phase (i.e., only the more stationary part of the listener's perception of the ABA- sequence–see Denham et al., 2013), while the Mill et al. (2013) also modeled the build-up process. That is, the possible perceptual objects are fixed in this model, whereas they are discovered on-line in Mill et al.'s (2013) model. The implementation is based on an assumed tonotopic space with three neural units receiving input from primary auditory cortex (A1). The three neural units correspond to the frequencies of the A and B tones and to the center frequency [(A + B)/2; marked by AB], and they represent three proto-objects (repeating A and B tones and the ABA tone pattern, respectively). Competition between these units is implemented without predictive processes. The model accepts tokenized ABA- input (as it was heavily specified for model these triplets) and it was validated on behavioral results.

#### Objects, sound organizations and network dynamics

In Wang and Chang's (2008) model objects are represented by synchronized oscillators. The oscillators are arranged into a two dimensional network with the dimensions of frequency and time (relative to the present). All units have local excitatory connections, and a global inhibitor receives excitation from all oscillators and in turn inhibits all units. The strength of activation of an oscillator thus depends on the external input, excitation from local connections (with lateral coupling modulated by tone repetition rate), and global inhibition (modulated by additive Gaussian noise). Each object is represented by a synchronized assembly of oscillators; different objects are represented by desynchronized assemblies. An oscillator is enabled only if it gets external simulation. If all the enabled oscillators are synchronized, the output state is integrated. The output state is segregated if all enabled oscillators of the same frequency are synchronized and oscillators of different frequencies are desynchronized. Therefore, the dynamics of the model can be interpreted as a competition between these two states, i.e., competition occurs between sound-organizations rather than objects. The main contributing factors to the competition are the randomness of the global inhibitor and lateral excitation. The width of the frequency dimension of the Gaussian function that describes lateral excitation decreases with increasing tone repetition rates. This biases the competition toward the segregated state with higher tone repetition rates and *vice versa*. Further, the range of variance of the global inhibitor increases with lower tone repetition rates, which widens the ambiguous range for slower sequences. This means that the model is parameterized in a way that it leads to the target experimental results.

In the model of Pichevar and Rouat (2007) the pre-processing stage organizes the oscillators into a two dimensional space (the first layer of the model). The first dimension is frequency. The second dimension is based on the assumption that the subcortical part of the auditory system extracts feature representations from the output of the cochlea. Therefore, a feature extracted for each cochlear channel is utilized as the second dimension. Two features were implemented in the model: amplitude modulation (yielding an “AMtopic” map) and averaged spectral energy (leading to a “spectrotopic” map), however, only one of these features was used at a time, chosen by the experimenter. Activity across the two-dimensional map of oscillators is governed by local excitatory and a global inhibitory dynamics. The activity of an oscillator (similarly to the oscillators in the model of Wang and Chang, 2008) depends on the external input, on the coupling between oscillators, on the amplitude of an intrinsic Gaussian noise, and on a global inhibitor. The weight between two oscillators (and hence their coupling) depends on the featural proximity of their external inputs: the weight is higher when the inputs are more similar. In the second layer, the binding between oscillators is determined. Each unit in this layer corresponds to one of the cochlear channels in the first layer. Therefore, the representation is one dimensional in this layer. Objects are represented on this second layer as synchronized units. The activation of a given unit depends on the temporal correlations between the frequency channels within the given cochlear channel. In the output of this layer the neurons belonging to the same object will be synchronized, and neurons from different objects will be desynchronized. Synchronization dynamically follows the temporal correlations within the frequency dimension. Global inhibition (a function of network activity) causes synchronized units from different objects to desynchronize. Competition between objects is thus determined by the dynamics of the second layer, which is governed by changes in temporal correlation and by global inhibition. The output of the model is a binary mask for each object, which indicates which channels are assigned to each object. In the actual implementation the number of objects (i.e., the number of masks) is pre-defined and set to be two, but can, in principle, be extended in order to segregate more objects.

Mill et al.'s (2013) model aims to simulate the perceptual dynamics of auditory stream segregation with a focus on multi-stable perception. Therefore, unlike the previous models, objects in this model are cyclically repeating patterns of discrete sound events. The first stage of the model continuously searches for repeating patterns embedded in the sequence, and forms representations that are evaluated according to their prediction of upcoming sounds in the sequence. Pattern links form probabilistically on the basis of event similarity; patterns made up of similar sounds are more likely to be discovered than those formed by dissimilar ones. Patterns do not necessarily include all sounds in sequence, and the stochastic link formation process allows the model to discover multiple interleaved embedded patterns within the sequence. Once a recurrent pattern is found it is considered to represent a candidate object. Object representations are implemented in the form of coupled excitatory and an inhibitory neural populations. In the second (competition) stage, the strength (activation) of object representations are affected by: (1) the rate of successful predictions they make, (2) the rate at which they are rediscovered (patterns discovery is a continuous process, the easier it is to discover a pattern, the stronger it is considered to be, effectively a representation of likelihood), (3) adaptation, self-excitation, and noise (these ensure stochastic switching between objects even without changes in the stimulation), and (4) inhibition from other objects if and when they predict the same sound event. The resulting activations in the model are bi-modally distributed, with objects having high or low states. The assumption is that at any moment in time, the object (s) with high state (s) are those that appear in perception. In contrast to the previous models in this section there is no global inhibitor, competition between object representations is mediated locally. Competition between objects that attempt to predict the same events leads to the emergence of compatible sets of objects, and the suppression of incompatible ones. Thus the model simulates the emergence of perceptual organizations without explicitly defining what they should be.

In Rankin et al.'s (2015) model, activation (neuron firing rate) of the three neural units represents the strength of the corresponding proto-object within the competition. The input from A1 is fed to all three units of the model, with tonotopy-based weights (high weight for the tone corresponding to the unit's best frequency, low for the opposite tone, and equal intermediate weight for the AB unit from both tones). Similarly to the model of Mill et al. (2013), each unit's activation depends on adaptation, self-excitation, and intrinsic noise. These ensure stochastic switching between integrated and segregated percepts even without change in the stimulation. Competition is implemented as inhibitory interactions between the three units. Inhibitory interactions are modeled in two different ways, yielding two different model variants. The first variant implements local—and lateral inhibition. Each of the three units produces instant local (self) inhibition as well as lateral inhibition to the other two units. The amount of lateral inhibition depends on the assumed tonotopic distance between the interacting units, decreasing with increasing frequency difference between A and B. In the second variant, global inhibition is implemented: the local and the lateral inhibitory factors are set to be constant. Adaptation in this case is implemented also as a slow decay of self-excitation. Model parameters were calibrated to match the behavioral data.

#### Output and model evaluation

Similar to the model of Barniv and Nelken (2015), the model of Wang and Chang (2008) has two possible outcomes: the output state is either integrated or segregated. Simulations exploring the effects of the main stimulus parameters of the auditory streaming paradigm (frequency difference and presentation rate) were reported to faithfully reproduce the three regions described by van Noorden (1975).

The output of the model of Pichevar and Rouat (2007) is the mask that can be used to reconstruct the original target sound. The model was tested for speech separation from intruding sounds (speech, pure tone, siren, telephone ring, and white noise). The authors compared their results to earlier computational ASA models as well as more general sound segregating models and reported that their model outperformed or was comparable to the models they considered.

The output of Mill et al.'s (2013) model is a time series representing the dominant object(s) at each point in time. For long auditory streaming sequences, human perception spontaneously fluctuates between the integrated and the sound segregated organizations. Simulations were reported to qualitatively reproduce the effects of stimulus parameters (frequency difference and presentation rate) on the proportions and phase durations of the different percepts, the difference between the duration of the first and subsequent perceptual phases (Pressnitzer and Hupé, 2006; Denham et al., 2013), the build-up of streaming, as well as the temporal dynamics of multi-stable perception observed in human data.

Although the number of proto-objects in Rankin et al.'s (2015) model is three, the model has only two possible outputs: integrated or segregated. That is, similarly to Barniv and Nelken's (2015) model, the output of this model represents the perceived sound organization (in contrast to the auditory object appearing in the foreground; as in Mill et al., 2013). Perception is classified as integrated if the activity of the AB unit is greater than the average of the A and B units. The model was evaluated in terms of its ability to simulate the distribution of typical phase durations, the proportion of each percept with regard to changes in the frequency difference, and the temporal coherence and fission boundaries. Performance of the two variants differed although both reported to qualitatively simulate the target data: the global inhibition variant provided better quantitative performance in the percept-proportion comparisons, whereas in the comparison with van Noorden's temporal coherence and fission boundaries, the local inhibition variant provided better quantitative results.

### Temporal coherence models

Models in this group are based on the notion that acoustic features of the sounds emitted by the same source recur together and thus grouping by temporal coherence will bind them together in veridical auditory object representations. As a consequence, there is seen to be no need to build alternative interpretations or to implement competition between them. In temporal coherence models, feature extraction is followed simply by grouping and clustering. The outcome is assumed to appear in perception with possible modulation by selective attention. The aim is to simulate the segregation of complex sounds using computations that can be plausibly implemented by cortical mechanisms. There are a number of models in this category; here we choose three exemplars that illustrate the key issues.

The model of Krishnan et al. (2014) is one of the latest computational models to exploit the idea of temporal coherence. The model takes as input an assumed ‘cortical representation’ of a complex auditory scene, computed by a pre-processing stage, and clusters the features that are temporally coherent. In this representation the features of the incoming sounds are represented in a multidimensional feature space derived from the spectrogram of the input sound. This representation is used in all of the models in this section, although the dimensions used can vary among them. The neuromorphic model of Thakur et al. (2015) is a computationally simplified version of Krishnan et al.'s (2014) model aiming only to segregate foreground and background, but in real-time. It introduces a formulation of selective attention, which works as an a-priori defined mask on the stimulus representation, selecting a subset of the coincidence matrices for computation.

Elhilali and Shamma's (2008) model is similar to those above. However, in this model clustering is based on the prediction of the next input, which is used to assign incoming sound events to one of the objects (in this case the number of objects is hardwired, and not learnt by the model). Because prediction is based on an autoregressive moving average (ARMA) model which is a stochastic process with additive noise, stochasticity can lead to the same stimulus generating different outputs.

The model of Ma (2011) also uses a similar feature representation and segregates sounds based on correlations between features. The principal difference lies in the features that are used and the way in which the representations are validated. The model is tested on tokenized inputs as well as on a speaker separation task.

#### Objects, sound organizations, and model dynamics

The inputs to Krishnan et al.'s (2014) model are the four-dimensional feature-representation explained above, together with a similarly organized two-dimensional pitch representation. These two input streams are initially analyzed in parallel and then later merged. The dimensions of the four dimensional feature-representation function are: time (in discrete steps), frequency, scale (defined in term of frequency component spacing, measured in cycles per octave), and rate (defined as temporal spacing, measured in cycles per second or Hz). The dimensions of the pitch representation are time (in steps identical to the other representation) and pitch. For grouping the features that are temporally coherent, coincidence matrices are calculated in a pairwise manner between the time sequences at each point on the frequency-scale plane and on the pitch axis using a sliding temporal window of ~30 to 500 ms duration. Coincidence is represented as a correlation matrix that evolves through time, and it is calculated separately for each rate between each of the frequency-scale channels and pitch channels. These coincidence matrices are then used to determine which of the channels are temporally coherent, i.e., strongly correlated across modulation rates. The calculated coincidence matrices are linked together into a large matrix in which the columns correspond to the pair-wise correlations between the frequency-scale and pitch channels, separately for each modulation rate at each time step.

The columns of this enlarged correlation matrix provide the input to a nonlinear principal component analysis (nPCA) stage, which is responsible for the feature grouping. The nPCA method serves as a mapping from multidimensional data to lower dimensions with minimal loss of information (Kramer, 1991). It is implemented in the form of a feed-forward auto-associative network with a hidden layer containing two units. The number of hidden units defines the number of objects that can be extracted, and is a static parameter of the model. The hidden layer behaves as a bottleneck that compresses the incoming information which is then mapped to an output layer, the same size as the input layer in order to reconstruct the input with minimal loss of information. The assumption here is that the units in the hidden layer represent significant features in the data. The two hidden units can therefore be interpreted as object representations. The outputs of this analysis are two “masks,” which contain a representation of each object at each time-step. They can be used to reconstruct the segregated sound sources from the multidimensional representation of the input.

In contrast with the nonlinear principal component analysis, when linear principle component analysis (PCA) is implemented (e.g., in the model of Elhilali et al., 2009a, not reviewed separately, as it is similar to Krishnan et al.'s (2014) model except for the type of PCA used), instead of the nonlinear version used here, the degree of freedom is lower. This is because PCA only finds objects that are anti-correlated, which is not necessarily the case in practice. In this model, the rank of the correlation matrix and the size of the eigenvalues determine the number of objects (i.e., it is assumed that objects with substantially lower eigenvalues than the rest are probably modeling noise). The model's outputs are the eigenvalues of the correlation matrix. Having two high eigenvalues (with ratio close to one) is taken as a sign of a segregated organization whereas one high eigenvalue reflects the integrated organization.

The model of Ma (2011) is also based on the temporal coherence principle. The input representation includes frequency, scale, pitch, and location features. The location feature is calculated by cross-correlating the spectrograms of the right left channels (the input here is a stereo sound). After feature extraction, grouping is done similarly to the Krishnan et al. (2014) model described above. The output is a set of masks that correspond to the detected objects, the number of which is a parameter of the model. Two types of mask formations are implemented. The first type is based on attentional signals, similarly to those in the model of Thakur et al. (2015). The other type uses supervised prior training on mask classification, i.e., to classify the target—and non-target masks. For this a support vector machine is implemented which categorizes the data into two clusters. Training is performed on mixed utterances and used in order to set up a segregating hyperplane in which the dimensions correspond to those in the input representation. Once it is calibrated, the support vector machine gives back the label of the mask (target or non-target) and the distance to the hyperplane.

The input representation in the earlier model of Elhilali and Shamma (2008) is similar to that of Krishnan et al. (2014). However, in this case it includes: time, frequency, pitch and scale, and excludes the rate feature. The model assumes that there are two clusters (~objects) or sound sources. The task in the grouping stage is therefore to assign the input at each time step to one of the two clusters. The output of the grouping stage is a 5 dimensional function with the same dimensions as the input, supplemented with rate which is derived from the input representation using multi-rate wavelet decomposition. The difference between this and the model of Krishnan et al. (2014) is that here the temporal dynamics are exploited in the grouping stage, while Krishnan et al. (2014) takes them into account in the feature extraction stage. Model-based predictions are used to determine the classification. Predictions are implemented through a stochastic latent variable (separately for each object with added noise). This variable represents the internal state of the given object as it evolves in time. The latent variable's current state is recursively estimated, using only the previous state of the object, and the most recent input-output decision. It is then used for predicting the upcoming input. The temporal coherence principle is exploited during the estimation of the next predicted output because locally smooth evolution is assumed for the feature representations. The estimation of the next output from each object leads to a prediction of its expected next input. This calculation is done using a first-order ARMA difference equation essentially describing stochastic connections between the input, output, and latent variable. At each step, the input is assigned to the object that provides the closest prediction. This clustering loop continuously operates on the incoming sound stream. However, the convergence of the algorithm is not discussed in this study.

#### Output and model evaluation

The main output of Krishnan et al.'s (2014) model takes the form of two masks. These can be applied to the sound sequence to resynthesize the segregated streams. The model was tested on sequences of tones and also on two mixed speech streams. The authors reported that the model segregated the high and low tones in the auditory streaming paradigm in accordance with van Noorden's (1975) integration/segregation regions, reproduced the bouncing effect (Steiger, 1980; Tougas and Bregman, 1985), segregated a new tone with distinct spectral features within a sequence of complex tones (Moore et al., 1986). The model was also tested on 100 mixtures of male-female pairs. When supplemented by lip movement information it was reported to improve the SNR of the selected stream by an average 6 dB.

he output of the model of Elhilali and Shamma (2008) is a 5 dimensional representation of the perceived streams. The spectrogram of a given object can be reconstructed by integrating the output along the scale and rate dimensions. The model qualitatively replicated the shape of the temporal coherence boundary found by van Noorden (1975) in the auditory streaming paradigm (however, there is an error in the paper, which shows the model generates the opposite classification to that reported by van Noorden, presumably a slip). The model can also segregate alternating /e/ and /ə/ vowels based on timbre and pitch. It shows sensitivity to onset asynchrony and, similar to the model of Krishnan et al. (2014), reproduced the crossing glide effect. Segregation of speech was tested on 400 mixtures and high resemblance was achieved between the representation of the original and the reproduced sounds. For vocoded speech the model output mimics the experience of hearing-impaired listeners, as expected, i.e., when the spectral and temporal resolution of the input was reduced the model segregated the input sounds less effectively.

The model of Ma (2011) was tested on tokenized input as well as on mixed speech. A simulation was performed on an ABAB sequence. A simulation was also run on complex harmonic sequences alternating between two fundamental frequencies. The model was reported to simulate behavioral results, i.e., it segregated the ABAB sequence and also the complex harmonics as expected from experimental studies (van Noorden, 1975). Speech sounds were also segregated, with the help of an attentional signal, similar to the one that was used in the model of Thakur et al. (2015). Comparisons between the two methods for mask formation showed no significant differences between them in this study.

## Summary and future directions

The three groups of models capture different aspects of the complex functions of ASA. Models based on Bayesian principles view ASA as a process assigning each segment of the input to one of the possible classes. The decision process ensures that the probability that the assigned class generated the segment is optimal, given the priors and the sound input. That is, these models assume that ASA implements an ideal Bayesian observer. There is evidence that perceptual decisions by human observers are indeed often close to those expected from an ideal observer (Kersten et al., 2004). These models are also inherently predictive, which allows them to continuously test their solutions against the sound input. However, the computational implementation of Bayesian principles is quite abstract (e.g., competition through adjustment of the priors), and thus it is not easy to see from these models, how they could be implemented by the human auditory system. In turn, neural models provide a more realistic account of competition between alternatives, and some of them also of the representation of objects. They can also include more detail of the type of effects influencing perception of multi-source acoustic scenes. That is, they are easier to expand to cover a wider set of ASA phenomena. On the other hand, compared to Bayesian models, they have more trouble in modeling the effects of prior knowledge, and are usually not fully implemented in neuro-computational form. Both neural and Bayesian models could be extended to deal with realistic input. However, feature extraction and feature binding processes cannot be regarded as being independent of the competition stage. That is, establishing some features (such as location or pitch) as well as binding them may depend on the currently dominant solution (perceptual organization); for results supporting this notion, see Gutschalk et al. (2005); Szalárdy et al. (2013). Therefore, the models in these categories would all require modifications to their two-phase structure (feature-extraction/grouping and competition), to allow interactions between the two phases. Temporal coherence models cut through this issue by offering a one-step solution in which coherence in multiple features establishes feature binding and object formation within a single process. They also utilize a different Gestalt principle (common fate) compared to that of most other models (similarity or smooth continuation). Further, there is evidence that temporal coherence is indeed extracted in the human auditory system (O'Sullivan et al., 2015; Teki et al., 2016). However, in contrast to neural and Bayesian models, existing temporal-coherence models don't offer a clear path for capturing higher-order regularities within the sound input, which are known to help auditory stream segregation (Dowling, 1973; Bey and McAdams, 2002; Bendixen et al., 2010; Szalárdy et al., 2014). Further, by eliminating the formation of representations for alternative sound organizations, they do not account for multi-stable stimulus configurations. On the other hand, while neither neural nor temporal coherence models are inherently predictive, some variants of both have prediction as an essential element (Elhilali and Shamma, 2008; Mill et al., 2013), thus adding the benefit of continuously testing their prediction against the actual input.

In general, the computational models reviewed above mainly implement algorithms for testing their utility in addressing specific aspects of ASA. This is not a reflection of the lack of effort for providing a complete model of ASA. Rather, this is due to the scarcity of firm evidence regarding the neural mechanisms involved in sound segregation, or auditory perception, in general. While some of the models are inspired by general oscillatory mechanisms thought to operate within the brain (e.g., Pichevar and Rouat, 2007; Wang and Chang, 2008), others are supported by neural evidence obtained with large-scale brain imaging methods (e.g., Ma, 2011; Krishnan et al., 2014), and yet others are mainly based on behavioral evidence (e.g., Mill et al., 2013; Barniv and Nelken, 2015). That is, the experimental evidence underlying these models is patchy at best. This also leads to quite different metrics in the measurement of their performance, such as SNR of the segregated streams (Nix and Hohmann, 2007; Krishnan et al., 2014), correlation between the original sound and the segregated sound feature representation (Elhilali and Shamma, 2008), similarity to results of selected behavioral experiments (Wang and Chang, 2008; Mill et al., 2013; Barniv and Nelken, 2015). The diversity of the measurement metrics clearly shows that the goals of these models differ from each other and, therefore, the performance of these models cannot be directly compared.

In fact, these models cannot be fully regarded as competing theories of ASA. Rather, they can be seen as covering different sub-processes of ASA, which may even be complementary. In essence, there are few true incompatibilities between the various models. Thus one possible future direction of the field may be to integrate (a subset of) these models, utilizing the strength of each in the next generation of models of ASA. For example, the notion of temporal coherence may be best suited to solving the feature-binding problem and the initial formation of sound chunks. In turn, neural models may best capture the linking of chunks across larger time-scales, the creation of proto-objects and their competition. Finally, Bayesian models may provide a predictive framework that allows the utilization of previous knowledge, and optimizes the perceptual decisions that are inherent in ASA. Such a composite model would also better fit the variety of Gestalt principles governing the formation of perceptual objects. One issue that is common to almost all models reviewed here is that whereas previous research has shown that temporal regularity/predictability helps auditory stream segregation (e.g., Andreou et al., 2011; Rajendran et al., 2013; Szalárdy et al., 2014), present models do not explicitly handle the temporal structure of sound sequences. Thus future models should explore how more nuanced temporal features beyond simple presentation rate can be taken into account in modeling ASA. It is likely that a hierarchical system will be needed to account for the findings related to showing the effects of higher-order structure on auditory stream segregation. Hierarchical structure and feedback from the later parts of the model may solve some of the paradoxes of ASA (such as the weakness of location cues; see Bregman, 1990; Kocsis et al., 2014). These features would allow future models to deal with realistic auditory scenes while also being testable on simple, theoretically important stimulus configurations.

## Author contributions

BS, SD, and IW participated in drafting the article, contributed to the writing of the paper, and gave final approval of the version to be submitted.

## Funding

This research was supported by the Hungarian Academy of Sciences (Lendület Project LP-36/2012).

### Conflict of interest statement

The authors declare that the research was conducted in the absence of any commercial or financial relationships that could be construed as a potential conflict of interest.
